# Group 3 pulmonary hypertension: Challenges and opportunities

**DOI:** 10.21542/gcsp.2020.6

**Published:** 2020-04-30

**Authors:** Michael McGettrick, Andrew Peacock

**Affiliations:** Scottish Pulmonary Vascular Unit, Golden Jubilee National Hospital, Glasgow, UK

## Introduction

**Figure 1.**
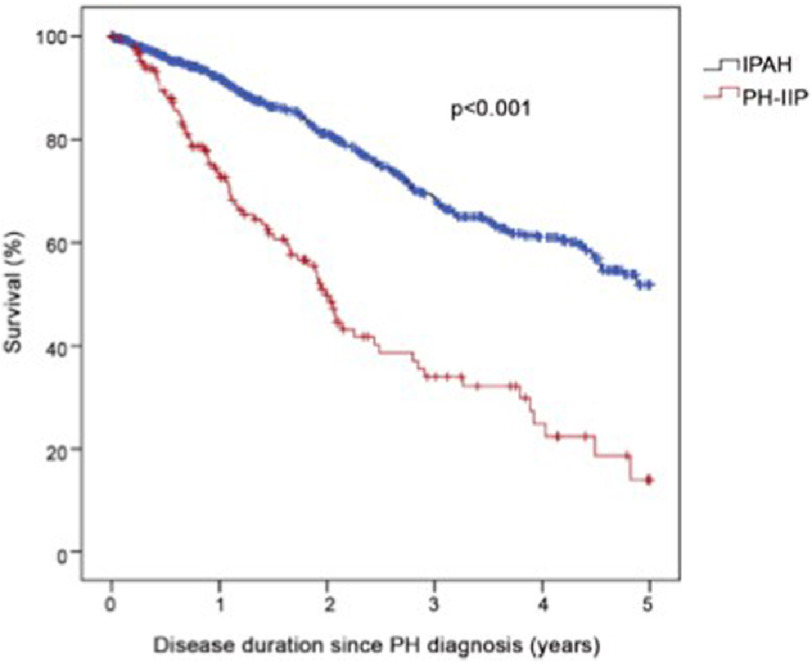
Survival comparison between IPAH patients (n= 798) and patients with PH secondary to hypoxic lung disease (n= 151). Survival was worse in the lung disease group (3-year survival 34% versus 68.6%, p = <0.001)^8^.

Pulmonary hypertension (PH) associated with hypoxia and lung disease, first identified as Group 3 in the 2008 Dana Point classification of PH^1^, is the second most common form of PH and is associated with increased morbidity and mortality (Figure 1)^2^. The most common lung diseases resulting in PH are chronic obstructive pulmonary disease (COPD), interstitial lung disease (ILD) and obstructive sleep apnoea (OSA)^3^ but is also associated with other diseases, such as cystic fibrosis^4^ and high altitude exposure^5^. Those with PH in the setting of obstructive or restrictive lung disease have worse outcomes, but it is not clear if the PH causes increased mortality or whether it is a marker for the severe end of the lung disease spectrum. Patients with Group 3 disease have a worse outcome than Group 1 IPAH (See Figure 1). Those patients with Group 1 disease pulmonary arterial hypertension (PAH) but with minor associated lung disease also suffer from worse outcomes^6,7^.

PH can occur in the healthy lung in response to low atmospheric oxygen, most commonly seen in populations who live at high altitude. In a similar mechanism to those with chronic lung disease, low alveolar oxygen (PAO_2_) causes hypoxic vasoconstriction^9^. Increase in growth factors and vasoconstricting hormones lead to increased hypertrophied vascular smooth muscle and increased vascular resistance^10^. As in lung disease, there is a variation in response to hypoxia. Kojonazarov et al screened 1430 patients who lived at altitude (>2500 m) in Kyrgyzstan, 26 of whom had PH^11^.

**Figure 2.**
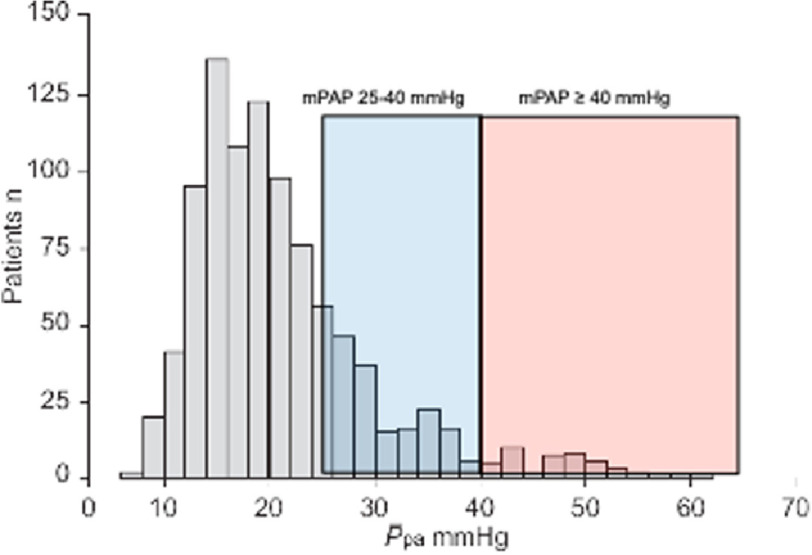
998 patients referred with COPD and respiratory failure. The prevalence of pulmonary hypertension wit mPAP>40mmHg was 2.7% of any cause and 1.1% secondary to COPD alone^14^.

PH in COPD is dependent on the severity of the underlying lung disease^12^ and prevalence depends on the definition of PH. Using the most up to date definition of PH from the latest World symposium, with mPAP >20 mmHg^13^, up to 90% of patients with Global Initiative for Chronic Obstructive Lung Disease stage IV have mPAP of >20 mmHg. 1–5% of patients have severe PH, with mPAP >35–40 mmHg^14^ (Figure 2).

**Figure 3.**
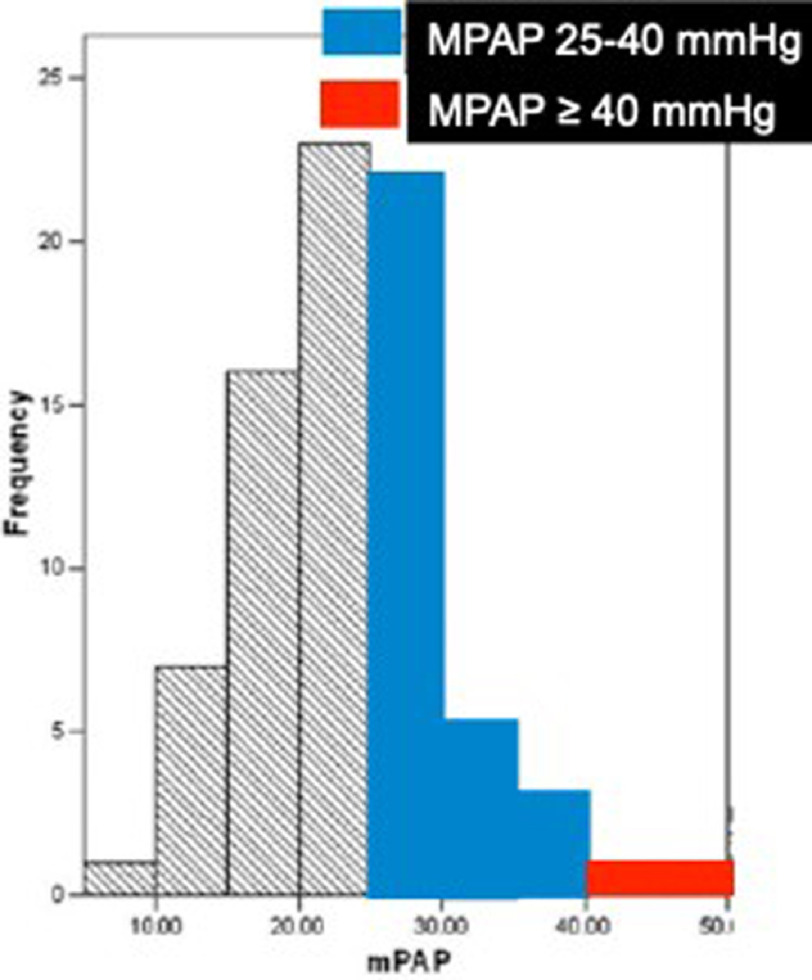
79 patients with idiopathic pulmonary fibrosis referred for lung transplant assessment. PH was found in 31.6% of patients^17^.

In the case of ILD, most of the literature uses an older definition of PH, with mPAP >25 mmHg and yet PH is still more common (See Figure 3). 8–15% of ILD patients are diagnosed with mPAP>25 mmHg at diagnosis, 30–50% with advanced ILD and >60% with end stage disease^15,16^. PH is associated with exacerbations of ILD and adverse outcomes, with the prognosis worse in those with ILD than in idiopathic pulmonary arterial hypertension (IPAH)^8^.

## Pathophysiology of Group 3 PH

The cause of pulmonary hypertension in hypoxic lung disease is multifactorial. In the healthy lung, the pulmonary circulation is a low-pressure system. Most of the resistance is a result of the natural reduction in caliber of the pulmonary vessels as they branch from the central arteries. When cardiac output increases during times of stress, the pulmonary vasculature is able to dilate to meet demand of higher blood volumes, and relatively under-perfused arteries are recruited. As a result, there is usually very little change in pulmonary artery pressure (PAP) during these times^14^.

In patients with chronic lung disease, however, there is a loss of blood vessels^18^ and, as such, there is reduced capacity to accommodate a higher cardiac output, leading to pressure increase. In addition, most chronic severe lung diseases result in periods of continuous or intermittent hypoxia. In contrast to the systemic circulation, there is contraction of pulmonary vessels in response to alveolar hypoxia^19^. In the early stages, this may be reversible with increased inspired oxygen concentration. However, with chronic hypoxia, there is release of vasoconstrictors such as endothelin and serotonin, which also serve as growth factors. This leads to intimal hyperplasia, and hypertrophy of the media, leading to increase vascular tone^20^.

This remodeling is only partially reversed with oxygen. Studies have shown an inverse relationship between PAP and PAO_2_^14,21,22^, with a higher PAP in those with lower PAO_2_. However, there is significant variability between patients’ responses to hypoxia. The reasons for this variability, which is also seen between species^23^, are not clear, but some patients have a greater increase in PAP when hypoxic, resulting in a higher risk of vascular remodeling over the longer term.

In chronic lung disease, it is traditionally thought that the changes in lung parenchyma lead to vascular changes, causing the pulmonary hypertension. However, there is a growing body of evidence to suggest that the underlying condition causing chronic lung disease can also cause vascular abnormalities, leading to rises in PAP that are out of proportion to the lung disease. There are several reasons why this is thought to be the case: firstly, histological samples of patients of idiopathic pulmonary fibrosis show evidence of vascular remodeling in areas of preserved lung^24^ and, secondly, severity of pulmonary hypertension does not correlate well with abnormalities in lung function, suggesting there is an additional mechanism leading to rises in pressure^25,26^.

Furthermore, there is increased expression of inflammatory mediators and growth factors in patients with pulmonary arterial hypertension^27^ that are also found in patients with lung disease associated pulmonary hypertension.

Endothelial cells detect hypoxemia, leading to reduced nitric oxide and increased endothelin-1 levels^28^. This then leads to contraction of the smooth muscle cells and increased cell proliferation by inhibition of anti-mitogenic factors, nitric oxide and prostacyclin, and by increasing the production of different mitogenic stimuli, such as platelet derived growth factor and vascular endothelial-derived growth factor. This proliferation of the vascular endothelium leads to increased vascular tone^29^. What is also relevant in group 3 PH, is that there is increased apoptosis of the pulmonary vascular endothelium in smokers, leading to a reduction in the NO synthetic enzymes^30^. This imbalance between natural vasodilators and vasoconstrictors creates an environment that is conducive to the development of pulmonary hypertension.

## Animal models of PH

The treatment of Group 3 PH has traditionally been to optimize treatment of the underlying lung disease and give long-term oxygen therapy to those who are hypoxic. The efficacy of pulmonary vasodilators in this group of patients is unclear. There have been mixed results from meta-analysis assessing the effects of vasodilators on exercise tolerance and quality of life^31,32^. More studies are required in order to establish the groups of patients who stand to most benefit from vasodilator therapy but the current advice is *treat the lung, not the pressure*.

Animal studies have been used to identify effective treatments in those with group 1 PAH. However, there has been little work done for those with group 3 disease. With the epidemic of pulmonary hypertension in the 1990’s, due to amphetamine based anorexigen medications, there was a surge in research into animal models. Initially, rats exposed to hypoxia were used. Although hypoxic vasoconstriction is also seen in all animals and birds living permanently at altitude, their responses to hypoxia are more blunted^33^.

Rats developed smooth muscle hypertrophy and right ventricular hypertrophy and were used in pre-clinical trials for decades^34^. These hypoxic animal models are similar, although less severe, than the disease process found in patients with pulmonary hypertension as a result of living at high altitude. However, there was a need to develop models that would facilitate further study of cell signaling pathways and the hope was that this would be facilitated by the advent of genetically modified mice.

The results of initial mouse models were disappointing because the degree of vascular remodeling and consequent PH was reduced in mice compared to rats for the same level of hypoxia. A number of animal models also demonstrated resolution of PH and RVH in response to normoxia^35^, which is clearly different from that seen in patients with chronic hypoxic lung disease because the pathophysiology is multifactorial, namely hypoxic vasoconstriction and rarefaction of the blood vessels^18,36^. Despite supplemental oxygen therapy making some haemodynamic improvements in hypoxic lung disease, it is rare for the pulmonary artery pressures to return to normal levels^37^.

Sugen (su5416) is a vascular endothelial growth factor blocker and was initially used to induce experimental pulmonary emphysema. However, when injected in to rats that were also hypoxic in the hope of developing a model for emphysema, the rats developed severe pulmonary arterial hypertension. Not only this, but the changes persisted when the rats were returned to normoxia^38^. The PH was also associated with obliterative vascular lesions, similar to those seen in human disease. These rats exposed to hypoxia then develop severe RV failure, similar to that seen in humans^39^. There have been multiple attempts to apply the same technology to mice, but these have largely been unsuccessful because of dissimilarity between mice and rats. In mice, the circulation is relatively unresponsive to hypoxia and they do not develop right ventricular failure^35^.

As it stands, there is no perfect animal for study of pulmonary hypertension, particularly in lung disease and further work is required to develop models similar to the human form of lung disease-PH^40^.

## Management of Group 3 PH

The management of pulmonary hypertension is dependent on the cause. In the case of PH secondary to high altitude, humans respond similarly to animal models of PH developed through chronic hypoxia: the PH is reversible when exposed to normoxia and sea level.

Trials of vasodilators have shown some promise in acute forms of altitude related PH: Ghofrani et al. showed that sildenafil reduced systolic pulmonary artery pressure during exercise and increased the maximal workload achieved at altitude^41^. Richalet et al. also showed improvements in pulmonary haemodynamics and exercise capacity when using sildenafil compared with placebo at altitude^42^. Bosentan has also been proven to improve haemodynamics at altitude. However, the mainstay of long-term treatment is oxygen therapy or, even better, descent to lower altitudes, which has the same effect^43^.

Treatment of PH in lung disease is a complex issue. Treatments should aim to optimize lung function and correct hypoxemia. The role of pulmonary vasodilators is more contentious. In patients with COPD, sildenafil and bosentan have shown improvements in pulmonary haemodynamics^31,32^, but this has not translated to improvement in exercise capacity and quality of life^44^. The evidence for use of pulmonary vasodilators in interstitial lung disease is even more disappointing. Randomised controlled trials have failed to show improvements in pulmonary haemodynamics^16,45^ and a meta-analysis showed no improvements in 6-minute walk distance (6MWD) or quality of life^32^.

Pulmonary hypertension is commonly associated with chronic lung disease, but severe PH is rare, defined as mPAP of ≥35 mmHg. Lange et al did show a survival benefit with vasodilators in patients with severe PH in lung disease, but not in the mild/moderate groups^46^. It has been shown that although pulmonary vasodilators do reduce NTproBNP in severe Group 3 PH, there was no significant increase in 6-minute walk distance. Response to therapy is different amongst the lung disease phenotypes, but, overall the evidence is that although pulmonary vasodilators are safe, there is a lack of effectiveness^47^.

## Management of Group 1 PAH with co-existing lung disease

Existing trials have shown efficacy of therapy in patients with isolated phenotypes of pulmonary hypertension. However, over recent years, the demographics of patients with PH has been changing, with an increasing age and with that the inevitable increase in comorbidities^48^. This makes exact diagnosis more challenging and application of clinical trial results more difficult in day-to-day clinical practice. Whilst the efficacy of pulmonary vasodilators in group I PAH has been clearly demonstrated, they have no use in group 3 disease and, it is not clear to what extent group I PAH patients with co-existing lung disease will respond to pharmacological treatment.

**Figure 4.**
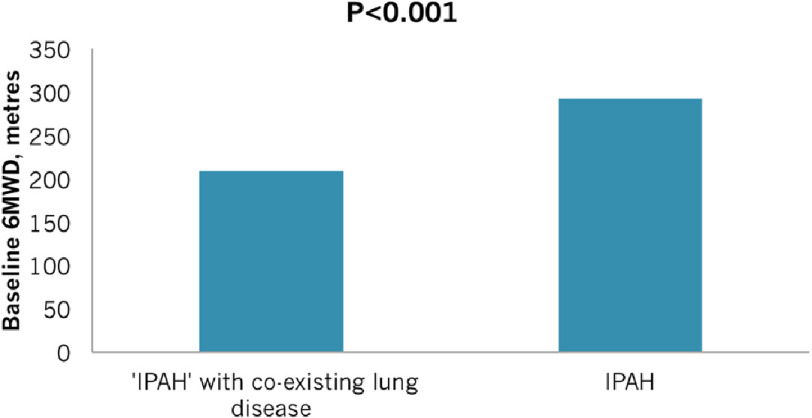
Baseline 6MWD comparison between those with IPAH alone (n = 482) and those with IPAH and co-existing parenchymal lung disease (n = 146). Those with parenchymal lung disease had worse 6MWD before treatment (p = <0.001), reproduced from [7].

Peacock et al.^7^ determined the response to treatment and survival in group-1 PAH patients with and without of radiological evidence of parenchymal abnormalities on CT Chest but with preserved lung function. 629 patients from the UK and Ireland were assessed. 482 patients were deemed to have ‘pure’ IPAH, without lung disease and 146 patients with radiological evidence of lung disease but preserved spirometry who had been diagnosed as Group 1 by the specialized PH centres of the UK and Ireland. The two groups had similar baseline haemodynamic characteristics, but with lower 6MWDs in those with lung disease (See Figure 4). As might be expected, diffusion capacity of the lung for carbon monoxide (DLCO) was noted to be severely reduced and significantly worse in those with parenchymal lung disease than with group I PAH alone.

**Figure 5.**
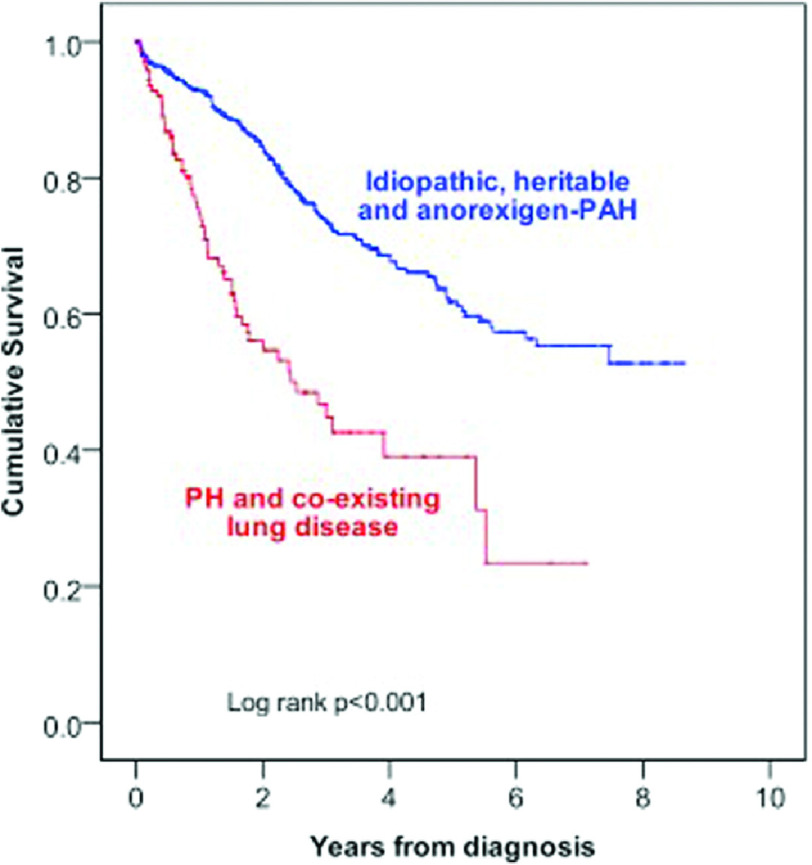
Survival analysis comparing those with IPAH alone (n = 482) and those with IPAH and co-existing parenchymal lung disease (n = 146). Those with parenchymal lung disease had worse overall survival, reproduced from [7].

Interestingly, the two groups had a similar response to treatment, as measured by 6 MWD, after 3 months, with no significant differences between the groups. However, survival was significantly worse in the patients with co-existing lung disease (see Figure 5).

At present, patients with group 1 PAH with *co-existing* lung disease should be treated with pulmonary vasodilators. Patients with PH *due* to hypoxic lung disease, even if severe, should have the treatment of their lung disease optimized and oxygen provided as needed and pulmonary vasodilators are unlikely to provide significant benefits.

## Future work in Group 3 pulmonary hypertension

In view of the poor prognosis associated with these conditions, and the lack of available specific therapy, further work is required to develop effective therapy. The World Symposium on Pulmonary Hypertension in Nice 2018 recommended better animal models of PH in both COPD and ILD, with parenchymal and vascular phenotypes developed in order to determine novel molecular targets^12^. In view of different aetiology, patients with Group 3 should be investigated differently in terms of molecular pathology and clinical course. Idiopathic interstitial pneumonia can be studied with chronic hypersensitivity pneumonitis and occupational lung disease. However, sarcoidosis should be studied independently, as should COPD^12^.

Establishing cellular and molecular causes of lung and vascular remodeling in cultured human lung tissue would be useful to identify therapeutic targets and to perform drug testing. Further research into biomarkers for group 3 disease and shared access to bio banks from existing registries would allow larger and more robust trials into therapeutic efficacy. More specific, “deep”, phenotyping of patients parenchymal and vascular disease using multiple imaging modalities may help to identify patient groups who would benefit from therapy^12^.

Utilizing more indices from the 6MWD, such as level of deoxygenation, Borg symptom score and heart rate recovery, is recommended to detect response to treatment. Cardiopulmonary exercise testing is also recommended to elaborate the distinction between respiratory and circulatory limitation.

With regards to specific future therapy, inhaled PH therapies are an attractive option as this may allow more effective ventilation-perfusion matching and minimise systemic side effects. Ideally, studies would focus on prevention, reversal or miminising vascular remodeling, rather than vasodilation. Studies should also target the vascular component in driving parenchymal abnormalities. The role of pulmonary rehabilitation is well established in parenchymal lung disease and in pre-capillary pulmonary hypertension, but further work is required on its efficacy in PH complicating lung disease.

## Conclusion

Group 3 PH is common and is associated with increased mortality rates compared to IPAH. The pathophysiology is different from that in other forms of pulmonary hypertension and different for each lung phenotype. However, currently, these patients are all treated as one phenotype. Animal models do not accurately reflect the cellular mechanisms seen in human disease and pulmonary vasodilators are not effective in these patients. More work is required to understand the changes leading to PH in patients with lung disease, which may lead to the identification of therapeutic targets that will improve outcomes.

## References

[1] Simonneau G, Robbins IM, Beghetti M, Channick RN, Delcroix M, Denton CP (2009). Updated clinical classification of pulmonary hypertension. J Am Coll Cardiol.

[2] Strange G, Playford D, Stewart S, Deague JA, Nelson H, Kent A (2012). Pulmonary hypertension: prevalence and mortality in the Armadale echocardiography cohort. Heart.

[3] Seeger W, Adir Y, Barberà JA, Champion H, Coghlan JG, Cottin V (2013). Pulmonary hypertension in chronic lung diseases. J Am Coll Cardiol.

[4] Fraser KL, Tullis DE, Sasson Z, Hyland RH, Thornley KS, Hanly PJ (1999). Pulmonary hypertension and cardiac function in adult cystic fibrosis: role of hypoxemia. Chest.

[5] Mirrakhimov AE, Strohl KP (2016). High-altitude Pulmonary Hypertension: an Update on Disease Pathogenesis and Management. Open Cardiovasc Med J.

[6] Poms AD, Turner M, Farber HW, Meltzer LA, McGoon MD (2013). Comorbid conditions and outcomes in patients with pulmonary arterial hypertension: a REVEAL registry analysis. Chest.

[7] Peacock A, Ling Y, Johnson M, Kiely D, Condliffe R, Elliot C (2020). Idiopathic Pulmonary Arterial Hypertension and co-existing lung disease: is this a new phenotype?. Pulmonary Circulation.

[8] Hoeper MM, Behr J, Held M, Grunig E, Vizza CD, Vonk-Noordegraaf A (2015). Pulmonary Hypertension in Patients with Chronic Fibrosing Idiopathic Interstitial Pneumonias. PLoS One.

[9] Xu XQ, Jing ZC (2009). High-altitude pulmonary hypertension. Eur Respir Rev.

[10] Voelkel NF, Gomez-Arroyo J (2014). The role of vascular endothelial growth factor in pulmonary arterial hypertension. The angiogenesis paradox. Am J Respir Cell Mol Biol.

[11] Kojonazarov BK, Imanov BZ, Amatov TA, Mirrakhimov MM, Naeije R, Wilkins MR (2007). Noninvasive and invasive evaluation of pulmonary arterial pressure in highlanders. Eur Respir J.

[12] Nathan SD, Barbera JA, Gaine SP, Harari S, Martinez FJ, Olschewski H (2019). Pulmonary hypertension in chronic lung disease and hypoxia. Eur Respir J.

[13] Galiè N, McLaughlin VV, Rubin LJ, Simonneau G (2019). An overview of the 6th World Symposium on Pulmonary Hypertension. Eur Respir J.

[14] Chaouat A, Naeije R, Weitzenblum E (2008). Pulmonary hypertension in COPD. Eur Respir J.

[15] Kimura M, Taniguchi H, Kondoh Y, Kimura T, Kataoka K, Nishiyama O (2013). Pulmonary hypertension as a prognostic indicator at the initial evaluation in idiopathic pulmonary fibrosis. Respiration.

[16] Raghu G, Nathan SD, Behr J, Brown KK, Egan JJ, Kawut SM (2015). Pulmonary hypertension in idiopathic pulmonary fibrosis with mild-to-moderate restriction. Eur Respir J.

[17] Lettieri CJ, Nathan SD, Barnett SD, Ahmad S, Shorr AF (2006). Prevalence and outcomes of pulmonary arterial hypertension in advanced idiopathic pulmonary fibrosis. Chest.

[18] Bignon J, Khoury F, Even P, Andre J, Brouet G (1969). Morphometric study in chronic obstructive bronchopulmonary disease. Pathologic, clinical, and physiologic correlations. Am Rev Respir Dis.

[19] Rudolph AM, Yuan S (1966). Response of the pulmonary vasculature to hypoxia and H+ ion concentration changes. J Clin Invest.

[20] Eddahibi S, Hanoun N, Lanfumey L, Lesch KP, Raffestin B, Hamon M (2000). Attenuated hypoxic pulmonary hypertension in mice lacking the 5- hydroxytryptamine transporter gene. J Clin Invest.

[21] Scharf SM, Iqbal M, Keller C, Criner G, Lee S, Fessler HE (2002). Hemodynamic characterization of patients with severe emphysema. Am J Respir Crit Care Med.

[22] Fayngersh V, Drakopanagiotakis F, Dennis McCool F, Klinger JR (2011). Pulmonary hypertension in a stable community-based COPD population. Lung.

[23] Grover RF (1965). Pulmonary circulation in animals and man at high altitude. Ann N Y Acad Sci.

[24] Colombat M, Mal H, Groussard O, Capron F, Thabut G, Jebrak G (2007). Pulmonary vascular lesions in end-stage idiopathic pulmonary fibrosis: Histopathologic study on lung explant specimens and correlations with pulmonary hemodynamics. Hum Pathol.

[25] Nathan SD, Shlobin OA, Ahmad S, Urbanek S, Barnett SD (2007). Pulmonary hypertension and pulmonary function testing in idiopathic pulmonary fibrosis. Chest.

[26] Barberà JA, Peinado VI, Santos S, Ramirez J, Roca J, Rodriguez-Roisin R (2001). Reduced expression of endothelial nitric oxide synthase in pulmonary arteries of smokers. Am J Respir Crit Care Med.

[27] El Chami H, Hassoun PM (2012). Immune and inflammatory mechanisms in pulmonary arterial hypertension. Prog Cardiovasc Dis.

[28] Bourque SL, Davidge ST, Adams MA (2011). The interaction between endothelin- 1 and nitric oxide in the vasculature: new perspectives. Am J Physiol Regul Integr Comp Physiol.

[29] Sandoo A, van Zanten JJ, Metsios GS, Carroll D, Kitas GD (2010). The endothelium and its role in regulating vascular tone. Open Cardiovasc Med J.

[30] Klinger JR, Abman SH, Gladwin MT (2013). Nitric oxide deficiency and endothelial dysfunction in pulmonary arterial hypertension. Am J Respir Crit Care Med.

[31] Chen X, Tang S, Liu K, Li Q, Kong H, Zeng X (2015). Therapy in stable chronic obstructive pulmonary disease patients with pulmonary hypertension: a systematic review and meta-analysis. J Thorac Dis.

[32] Prins KW, Duval S, Markowitz J, Pritzker M, Thenappan T (2017). Chronic use of PAH-specific therapy in World Health Organization Group 3 Pulmonary Hypertension: a systematic review and meta-analysis. Pulm Circ.

[33] Swenson ER (2013). Hypoxic pulmonary vasoconstriction. High Alt Med Biol.

[34] Stenmark KR, Meyrick B, Galie N, Mooi WJ, McMurtry IF (2009). Animal models of pulmonary arterial hypertension: the hope for etiological discovery and pharmacological cure. Am J Physiol Lung Cell Mol Physiol.

[35] Taichman DB, Mandel J (2013). Epidemiology of pulmonary arterial hypertension. Clin Chest Med.

[36] Colvin KL, Yeager ME (2014). Animal models of pulmonary hypertension: matching disease mechanisms to etiology of the human disease. J Pulm Respir Med.

[37] Weitzenblum E, Sautegeau A, Ehrhart M, Mammosser M, Pelletier A (1985). Long- term oxygen therapy can reverse the progression of pulmonary hypertension in patients with chronic obstructive pulmonary disease. Am Rev Respir Dis.

[38] Taraseviciene-Stewart L, Kasahara Y, Alger L, Hirth P, Mc Mahon G, Waltenberger J (2001). Inhibition of the VEGF receptor 2 combined with chronic hypoxia causes cell death-dependent pulmonary endothelial cell proliferation and severe pulmonary hypertension. FASEB J.

[39] Bogaard HJ, Natarajan R, Mizuno S, Abbate A, Chang PJ, Chau VQ (2010). Adrenergic receptor blockade reverses right heart remodeling and dysfunction in pulmonary hypertensive rats. Am J Respir Crit Care Med.

[40] Gomez-Arroyo J, Saleem SJ, Mizuno S, Syed AA, Bogaard HJ, Abbate A (2012). A brief overview of mouse models of pulmonary arterial hypertension: problems and prospects. Am J Physiol Lung Cell Mol Physiol.

[41] Ghofrani HA, Reichenberger F, Kohstall MG, Mrosek EH, Seeger T, Olschewski H (2004). Sildenafil increased exercise capacity during hypoxia at low altitudes and at Mount Everest base camp: a randomized, double-blind, placebo- controlled crossover trial. Ann Intern Med.

[42] Richalet JP, Gratadour P, Robach P, Pham I, Déchaux M, Joncquiert-Latarjet A (2005). Sildenafil inhibits altitude-induced hypoxemia and pulmonary hypertension. Am J Respir Crit Care Med.

[43] Kojonazarov B, Isakova J, Imanov B, Sovkhozova N, Sooronbaev T, Ishizaki T (2012). Bosentan reduces pulmonary artery pressure in high altitude residents. High Alt Med Biol.

[44] Park J, Song JH, Park DA, Lee JS, Lee SD, Oh YM (2013). Systematic review and meta-analysis of pulmonary hypertension specific therapy for exercise capacity in chronic obstructive pulmonary disease. J Korean Med Sci.

[45] Corte TJ, Keir GJ, Dimopoulos K, Howard L, Corris PA, Parfitt L (2014). Bosentan in pulmonary hypertension associated with fibrotic idiopathic interstitial pneumonia. Am J Respir Crit Care Med.

[46] Lange TJ1BM, Seiler I, Arzt M, Pfeifer M (2014). Outcome of patients with severe PH due to lung disease with and without targeted therapy. Cardiovascular Therapeutics.

[47] Brewis MJ, Church AC, Johnson MK, Peacock AJ (2015). Severe pulmonary hypertension in lung disease: phenotypes and response to treatment. Eur Respir J.

[48] Hoeper MM, Gibbs JSR (2014). The changing landscape of pulmonary arterial hypertension and implications for patient care. Eur Respir Rev.

